# Recruiting Black Men Who Have Sex with Men and Women (BMSMW) in an Urban Setting for HIV Prevention Research

**DOI:** 10.4172/2155-6113.1000408

**Published:** 2014-12-30

**Authors:** Gerald Asare Bempong, Hema C. Ramamurthi, Jason McCuller, John K Williams, Nina T Harawa

**Affiliations:** Charles R. Drew University of Medicine and Science, Los Angeles, CA, USA

**Keywords:** Black/African American, MSMW, Bisexuality, Recruitment, HIV, Internet and research

## Abstract

**Objectives:**

Concerns related to stigma and confidentiality limit HIV-related study recruitment and retention of Black men who have sex with men and women (MSMW). This paper examines lessons learned from recruitment strategies utilized in enrolling 437 participants between 2007 and 2011 for a randomized controlled trial to test a culturally specific HIV risk-reduction intervention for Black MSMW.

**Methods:**

Interested respondents completed a brief screener and participants completed surveys at baseline and at post, 3 and 6 months follow-up. Recruitment patterns were assessed by examining the source of study information reported when respondents were asked how they learned about the study. Chi-square tests were then conducted to examine differences in the distribution of participants by self-reported HIV status, age group and socio-economic status (SES) for each type of study information source.

**Results:**

Regardless of HIV or SES, study respondents were more likely to have received information about the study through a service agency than from other sources. Participants ages 30–49 and 50+ years were most likely to have learned about the study from an agency (34.9% and 27.0%, respectively) or another participant/friend (20.1%, 23.1%) and least likely to have found out from bus (0.8%, 0.0%) or radio (1.3%, 0.0%) advertisements; whereas younger participants were more likely to have heard about the study through a friend/participant (23.4%) than an agency (15.4%). Although, 14.1% of participants’ ages less than 30 years reported the Internet as their source of study information, this compared to just 2.7% and 4.8% for participants in the 30–49 and 50-plus age groups.

**Conclusions:**

While agencies and referrals from personal networks appear to be the most significant recruitment source for potential HIV research participants, there is evidence that Internet based tools may enhance recruitment, particularly among younger Black MSMW.

## Introduction

Starting from the premise that obtaining reliable and generalizable research findings requires effective recruitment and retention of the target population, low minority participation rates in health-related research can reduce study applicability and efficiency [1–3]. Fear and distrust towards medical research has been fostered by studies in which minorities were deceived or taken advantage of, [4–7] and by individual and community experiences of poor or discriminatory healthcare encounters. This distrust, together with lack of reading level appropriate and language-specific information, complex informed consent processes, [8] and limited access to healthcare and transportation [9] has contributed to low research participation rates [2,10]. The situation becomes even more challenging when research is focused on sensitive and traditionally stigmatized sexual behaviors in minorities.

In response to the Federal requirements designed to foster equitable selection of research participants, many researchers have implemented culturally, linguistically and socially relevant recruitment strategies [11]. Despite these efforts, recruiting specific subgroups such as minorities from large metropolitan areas remains challenging [12]. For example, low-income urban neighborhoods present additional challenges of increased resident turnover and reduced interest in prevention interventions, particularly when individuals lead a day-to-day existence with regard to food and shelter [12]. In addition, community-specific factors can present additional recruitment obstacles for studies on issues such as HIV/AIDS [10,13]. For example, in African American communities, churches often serve as a recruitment resource for research participants [14]; yet there is limited support when studies target risky sexual behavior and sexual minority populations [15,16]. Fear of ostracism, ridicule and alienation from within the Black community [17,18] complicates efforts to seek Black MSM from general community networks and social settings [19]. Conspiracy beliefs about HIV being a man-made disease to harm people of African descent also dissuade some Black men from engaging in HIV research [20,21].

The high HIV incidence among Black MSM [22–26], surveillance data indicating that they compose nearly half of all cases of HIV among Black people, and their potential role in a substantial but unknown number of cases among Black women [25,26] have brought attention to the need for better engagement of these men in HIV risk-reduction and treatment efforts. Strategies such as employing racially/ethnically matched research staff, including representatives from the target population in the research development process, building collaborations with service agencies and key stakeholders who work with and have the population’s trust [27,28] developing culturally relevant materials and communication strategies [6,27,29] as well as recruiting from gay venues [22,30–32] and through gay media [29] have been successful in reaching minority MSM. However, many of these efforts have focused on Black MSM in general, with little information specific to engaging those who also have sex with women (MSMW) – a unique subgroup of MSM. HIV-positive Black MSMW compose 31% of all Black MSM diagnosed with HIV in 2011 [33]; hence they are important targets for addressing the HIV epidemic. Furthermore, because many Black MSMW do not construct their personal identities around their same-sex behavior [34,35] and reside within communities with few gay venues, [36] the above-mentioned strategies may have limited success or yield non-representative samples.

A few researchers have implemented ethnographic mapping techniques in order to conduct targeted sampling in social and sexual spaces where MSMW congregate [37,38]. To date, however, we are not aware of a published comparative analysis of the relative effectiveness of various recruitment venues and methods. To address this gap and inform future efforts to recruit Black MSMW for HIV-related research, we describe and examine differences in the yield of the various outreach and recruitment strategies we employed in order to recruit African American/Black MSMW for the “Men of African American Legacy Empowering Self ” (or *MAALES*) Project – a randomized trial of a new behavioral HIV risk-reduction intervention [39]. We also analyzed data on those screened by HIV status, strata of socioeconomic status (SES), and age group in order to assess what outreach venues and approaches might be best for reaching Black MSMW within these specific sub-groups. This analysis was a tertiary objective of the study – one not originally proposed but conceived early in the implementation phase.

## MAALES project trial overview

The MAALES Project trial tested a new intervention designed specifically for Black MSMW, entitled Men of African American Legacy Empowering Self or MAALES, against a control condition involving a single client-centered HIV risk-reduction session. Briefly, the three-week, six-session (12 hours total) small-group intervention was developed with the collaborating agencies and informed by community advisory board members and extensive formative research. Intervention objectives, format, and activities were guided by the Theory of Reasoned Action and Planned Behavior, Empowerment Theory, and the Critical Thinking and Cultural Affirmation Model – an Afrocentric model developed by one of our community collaborators and based on the Social Cognitive Theory [40]. To best mirror eventual intervention dissemination, many of the intervention sessions were held at the partner agencies. A more detailed description of the intervention and the theory on which it is based can be found in the article by Harawa et al. [41].

Assessments were conducted at baseline, immediately post intervention and at 3 and 6 months post intervention. Eligibility criteria included self-identification as a Black/African American male, 18 years of age or older, having engaged in some type of sexual activity with both a male and a female in the prior 24 months, and not having engaged in another HIV prevention program in the prior six months. HIV-positive, HIV-negative, and unknown status men were all eligible to participate and HIV testing was encouraged but not required for study participation. Although MAALES focused on African American communities and norms, our Community Advisory Board (CAB) indicated that the intervention might resonate with Black men, regardless of their ethnicity. Hence, eligibility was open to men who identified as Black or African American.

## Methods

### Outreach and recruitment strategies

During the initial study development phase in 2005, a Community Advisory Board (CAB) with 7 members selected for their expertise and knowledge about the life, culture and practices of African American men living in Los Angeles was created. Most CAB members were known to a self-identified Black MSM research assistant through his extensive community contacts and HIV research experience. Additional CAB members were recruited by the investigators and by other CAB members through their own professional and personal networks. All CAB members were Black men and most self-identified as gay, bisexual or same gender loving. CAB members provided input on the content and format of recruitment materials and curriculum and advised on locations and events for reaching BMSMW. Later, they were encouraged to share study information with potential participants. Our community partners -- three local community based organizations (CBOs) that offered a range of HIV and non-HIV-related services -- also provided extensive input on all aspects of the study development.

During the formative phase, we conducted focus groups and in-depth interviews with Black MSMW, women who were or had been sexual partners of BMSMW, and service providers who interacted with Black MSMW in various settings. We asked participants about barriers for Black MSMW to participate in a small-group HIV risk-reduction intervention and their suggestions about recruitment approaches and locations. Details of the focus group findings can be found elsewhere [40,41] and further information on the intervention development and content can be found in Williams et al. [42].

### Conducting outreach and recruitment

All recruiters were selected based on their ability to both interact and do outreach with Black MSMW. In addition, most recruiters had prior prevention or outreach experience with this population. We looked for recruiters who were comfortable in both general African American and gay/bisexual communities and venues and who could establish rapport quickly. Also essential was that our recruiters were perceived as nonjudgmental and trustworthy – unlikely to share potential respondents’ private information. Our CBO partner sites were the focus of some of our recruitment efforts. They were non-gay-identified agencies that provided both HIV and non-HIV-related services – factors that likely increased Black MSMW’s general comfort level with accessing their services. Some other recruitment sites provided services unrelated to HIV, health, or sexual orientation (e.g., job training or access to general relief). To minimize study associated risks and maximize privacy, outreach was often conducted at these sites without the agency staff members being told that our target population was MSMW. Field team members were trained in various aspects of recruitment and compliance with human subjects’ regulations, including: 1) maintaining personal safety and boundaries, 2) employing effective communication strategies, 3) describing the project appropriately, 4) screening for eligibility, and 5) maintaining privacy and confidentiality.

Recruitment for the intervention phase started in July 2007 and ended in May 2011. Representatives from two academic institutions and our three CBO partners formed the field team. Because the intervention was designed to be facilitated by ethnically matched men, most of the recruiters were themselves self-identified African American men. However, not all were MSMW. Over the course of the project, we had male recruiters who identified as homosexual, bisexual and heterosexual. We also had two women, one African American and one African-born who carried out a significant amount of recruitment. Occasionally, the female Indian-born Project Director conducted outreach and recruitment as well. Female recruiters have been successful in other research with Black MSM [28] and our focus group participants reported that some Black MSMW might prefer to interact with a female than a male recruiter in public places. They and CAB members further indicated that some MSMW might be uncomfortable if approached in such settings by male recruiters who they perceived to be effeminate. Our community-partnered research approach was helpful for negotiating such gender stereotypes and concerns regarding emasculinization while working to recruit this population, as many field staffs were CBO members who had successfully engaged MSMW in the past.

A recruitment goal of at least 8 new enrollees per month was established early in the study. Recruiters generally worked in teams to maintain morale and mitigate rejection fatigue and boredom in the field. They completed weekly recruitment logs that included information on recruitment dates, times and locations with an estimate of the number of potentially eligible men present and contacted at each. This information was used to plan future recruitment activities. Although recruitment activities occurred throughout Los Angeles County (LAC), the team focused in the South and Central regions – the areas of LAC that have the largest populations of African Americans.

### Recruitment materials and methods

We used a wide range of recruitment materials including flyers, postcards, tri-fold brochures, matchbox style condom packets, bus placards, social media and the internet. Language and content (i.e., images and wording) varied to facilitate the recruitment of diverse types of men -- younger men, older men, men at gay clubs, and men in public places that included women. We used language that encouraged participation of “sexually active” African American men, with some flyers specifying those engaging in bisexual behavior. The recruitment scripts and all recruitment materials used also emphasized confidentiality and the potential for cash compensation. Following CDU IRB regulations, incentive amounts were not printed on recruitment materials but were provided during the screening process or discussions with recruiters. All materials used by our research team were approved by the institutional review boards (IRBs) within the two collaborating academic institutions. All recruitment materials incorporated the MAALES project logo. Most also included a black and red color scheme and the Adinkra (West African) symbols used in the intervention and photographs of Black men.

Distribution of promotional materials involved both active and passive approaches. During active recruitment, recruiters handed out flyers to Black men and explained the project to those indicating interest. During passive recruitment, recruiters displayed materials in our collaborating agencies, and in other venues that were frequented by African American men. Locations are described in Table 1. Staff also distributed small packets that included two condoms, lubricant, a mint candy and a small flyer or left them in jars at Black barbershops. The packets left in the barbershops did not include lubricant packets, as experienced staff members indicated that the presence of lubricant may generate homophobic discourse among customers.

Media outreach included local community newspaper, radio, Internet, and public transit advertisements. We ran weekly advertisements in a small number of Black community newspapers. The longest run was 3 years (at a total cost of $7,800). We also purchased advertisements on a popular radio station targeting African Americans that ran for 4 months ($3,200). Advertisements were also run inside of public buses with high African American ridership for 2.2 years ($24,000) and on bus stop benches for approximately 6 months. Study staff created a website and a Facebook page for the study and weekly announcements were placed on craigslist.com with the title, “Voluntary UCLA/ CDU Research Study for Black Men” for over 2 years. It consistently yielded a few calls per month at minimal cost (i.e., $25 per ad). Attempts were also made to recruit participants through messaging in online chat rooms and dating websites; however, site-specific restrictions and limited response led to our discontinuing this approach. Paid banner advertisements on a major newspaper and a black gay dating site also yielded fewer than five calls over a couple of months and were discontinued.

We sought referrals from our collaborators and various community based agencies and health clinics. We contacted providers and staff directly via regular and electronic mail, telephone, and through presentations. Although the Investigators or the Project Director sometimes made initial contact with agencies, it was the field team’s responsibility to replenish materials and remind the agency staff about the project. Additionally, we utilized respondent-driven sampling approaches [43] by giving interested study participants 3 coupons that could each be redeemed for $15 cash if they gave them to friends who later enrolled in the study. We also provided a brief training on how to make such referrals.

As part of the recruitment drive, study staff also hosted four events and participated in a variety of community events. In Year 1, we organized a kick-off party at a local club to introduce the project to the target population and an informational breakfast at the lead university to introduce it to providers. In later years, we also conducted two one-day HIV testing events targeting the clientele of several local barbershops serving African American men. These events provided opportunities to reach potential participants and build positive relationships with the barbers themselves. The MAALES Project also hosted booths or did outreach on foot at a variety of street festivals and health fairs. A popular activity in these settings was an HIV/STD knowledge quiz that offered attendees the opportunity to win small prizes (e.g., study pens or magnets) and encouraged them to dialogue with MAALES team members.

### Screening and enrollment

Interested individuals were screened by a recruiter in the field or over the telephone. In addition to determining eligibility, potential participants were also asked how they found out about the project. In our efforts to determine where best to disseminate study information, respondents who reported learning about the study through more than one source were grouped into the most specific source/location they could identify. Once respondents were screened eligible, they were scheduled for the informed consent process and baseline interview. Data were collected using an audio computer-assisted self-interview (ACASI) that assessed socio-demographics, HIV testing, sexual- and drug-risk behaviors, and other covariates.

### Data analysis

Data analysis was conducted using SPSS and SAS 9.3. Cross tabulations were calculated to examine recruitment patterns by how those screened found out about the study and to examine differences in information source by age group, HIV status, and socioeconomic status (SES) among study participants. Two-way chi-square tests were conducted to assess differences in the distribution of participants by self-reported HIV status (HIV+, HIV-, unknown), age group (<30, 30–49, and 50+ years), and socioeconomic status for each type of study information source. Fisher exact tests were reported for cross tabulations with cells less than 6. We used a composite SES measure that encompassed highest educational level completed (<High school/ GED=0, High School/GED=1, College or higher=2), monthly income (<$1,000=0, $1,000–1,999=1, $2,000–2,999=2, ≥$3,000=3), and current employment status (Yes = 1, No = 0). Income was based on individual income from all sources (including public assistance), before taxes. The sum of these scores for each participant was then used to categorize him into 3 SES strata: low=0–1, middle=2, higher middle=3+.

## Results

Table 1 provides the total number and relative distribution of the logged field-based outreach activities. Nearly half of all outreach activities were carried out at non-governmental agencies (27%) and private businesses (18%). A substantial amount of recruitment activity (21%) was also carried out through street activities (on sidewalks, street corners, college campuses, the beach, parks); while a relatively small number (7%) were carried out in transit areas (buses/train stations), bars/club (5%) and sex venues (4%); however, each of these sites still involved greater than 25 activities.

The study recruitment target of 8 eligible participants per month was on average exceeded. In Year 1, which included only 6 months of recruitment, 46 participants were enrolled, while 126, 136 and 105 participants were enrolled in years 2–4, respectively. In year 5, which included the last 5 months of the study, only 24 participants were enrolled. Thus, for the 47 months of recruitment, 437 men were recruited and enrolled, which exceeded our original target of 376. Throughout the years, enrollment tended to be low in November and December. This periodic drop in enrollment may be related to waning interest from potential participants and staff vacation time during the holidays.

A total of 862 interested respondents completed the screening. Out of those screened, 491 (57%) were deemed eligible, 437 (51%) were enrolled, 381 (44%) were randomized, and 291 (34%) were retained. Hence, nearly twice as many individuals were screened as were enrolled and three times as many were screened as were retained over the study course. Outcome analyses were based on whichever follow-up assessment was completed last, the 3 or 6 months [39]; therefore, we base our overall retention on those completing either of these post assessments. Table 2, shows the distribution of information source for those screened (respondents) and shows these distributions in terms of the percentage of respondents who screened eligible, the percentage of screen eligibles who enrolled, and the percentages of enrollees who were retained.

Among those screened, 20.1% reported learning about the MAALES Project through service agencies. A total of 21.3% said they had seen or received a flyer (16.0%) or spoken to a recruiter (5.3%) but were unable to recall where or how this occurred and, therefore, could not be grouped into a specific recruitment location. Friends (who may or may not have been participants) and identified study participants were also a significant source of referrals, contributing 19.8%. Outreach efforts in bars and clubs, yielded just 11 individuals who were screened and 4 who enrolled. Individuals in these settings ignored the materials, expressed resistance, or expressed interest and engaged with recruiters but never followed up for screening. Eligibility at screening ranged from a high of 80% for those learning about the study through agencies to a low of about 36% for individuals who found out about the study at a bar/club or through newspapers or flyers. Their reasons for ineligibility varied. The percentage of eligible enrollees retained at follow up ranged from 56% to 100% across sources; however, a chi-square test of retention differences across information sources was not significant (p=.60).

### Study participant description

Participants’ mean age was 42.6 years (SD=10.4). The majority reported being unemployed (45.5%) or unable to work/disabled, 32%. A monthly income of less than $1000 was reported by 56%, $1000–1999 by 22%, $2000–4999 by 15%, and $5000 or more by just 6.6% of participants. A little under half of study participants reported having tested HIV positive (47%) and 41% reported last testing HIV negative. The remainder reported having never tested or last receiving an indeterminate or inconclusive result (9%); 3% refused or did not answer. The majority of participants (58%) reported having a high school diploma or GED; 17% had not completed high school; 18% had obtained a two year associate degree or certificate, and 7% had a college or a higher degree. Nearly two-thirds of participants (61%) self-identified as bisexual; 15% identified as homosexual or gay and an additional 14% as heterosexual. Finally, 1.6% and 6.3% identified as same gender loving and down low respectively, while 1.6% refused to answer or chose “other”.

Table 3 displays differences in recruitment source by HIV status. The 204 HIV-positive participants were utilized as the referent group and compared separately to the HIV negative and unknown status men. HIV-positives were more likely to be recruited into the study through an agency compared to HIV-negatives (42.1 vs. 19.4%; p<.0001) and men of unknown HIV status (42.1 vs. 19.5%; p=.006). HIV-positive MSMW were less likely to be recruited through bus advertisements than were HIV-negative MSMW (3.9 vs. 16.1%; p<.0001). In addition, those recruited through newspaper advertisements comprised a greater portion of HIV negatives (p<0.0001) than those of unknown HIV status (p=0.034) when compared to those who were HIV positive (8.9 and 7.3 vs. 0.9%).

Eighty percent of participants were either low- or mid-level SES. Table 4 shows the comparison of low SES participants to those of middle or upper middle SES. The only significant difference found was somewhat surprising, a higher portion of middle (p=0.067) and upper-middle SES participants (p=0.022) reported bus advertisements as their source of study information than did low-SES participants (10.7 and 13.6 vs. 5.4%). Despite targeted efforts to engage men of younger age groups, more than 85% of participants were over the age of 30. Table 5 displays a comparison of the referent group (age 30–49) to those who were under the age of 30 as well as those aged 50 and over. Although no significant difference in recruitment source were observed for older participants, differences were observed in the younger age group. The under 30 age group was less likely to be recruited through an agency (15.6 vs. 34.9%; p=.002) than those age 30–49; however, they were more likely to hear about the study by way of the Internet (14.1 vs. 2.7%; p<.0001) or Radio Advertisements (3.1 vs. 0.4%; p=.038). A Friend/ Participant, Agency, and the Internet were, respectively, the leading information sources for young participants.

## Discussion

Our findings indicate that service agencies played a key role in recruitment, both through our community partners and their contacts at other agencies and through serving as venues for direct and indirect recruitment. This finding is consistent with other studies that have examined effective methods of recruiting specific populations [44– 47]. Our results indicating that study participants recruited from agencies were more likely to enroll and complete the study protocol could be explained by the fact that many of these individuals were already receiving ongoing services and had developed habits that foster consistent engagement or that they may have been more motivated than other recruits. Furthermore, it is plausible that people already receiving services from an agency would be more inclined to participate in research recommended by that agency. HIV-positive clients may also have had prior research experience encouraging them to take part. Although outreach was not limited to those agencies providing HIV services, we did outreach to a wide variety of HIV service providers, including three that were adjacent to our main study office. The fact that community health and social service agencies tend to serve people of lower SES may also have contributed to the underrepresentation of middle class and affluent SES men in this study. Thus, outreach through these service agencies should not displace other strategies that may reach more affluent men.

The importance of positive word of mouth from participants and friends is reinforced by the high referral rates to the MAALES project. Recruitment approaches that have successfully built on the networks of study participants include respondent-driven [48] or snowball sampling; both methods that use members of the desired population subgroup as seeds to identify other subgroup members [49]. Although we also made available to participants incentives for making successful referrals, only a handful of our study participants sought the compensation for their referrals to the study. Others referred men without seeking the incentive. More research into what motivates individuals to refer or not refer others into research on stigmatized populations such as this one may help encourage even more referrals into future studies. However, it seems clear that participants would only refer others if their own experiences as study participants were positive.

Traditional, low-technology recruitment approaches, such as distribution of flyers and smaller palm cards continue to yield success. However, although interested respondents frequently mentioned receiving or seeing these hard-copy materials, many could not provide the exact location or circumstance under which this occurred. This lack of detail limited our ability to determine the most efficient venues for distribution of flyers and other “small media”. We even attempted color-coding flyers for different types of recruitment venues; however, participants did not always remember the flyer color or have it with them when they called. Because of the more widespread use of mobile devices with both geopositioning services and applications that can instantaneously scan coded features, the placement of Quick Response (QR) codes on outreach materials [50] can now be used to better determine which outreach materials are responded to and where this occurs [51].

Our participation in community events and advertisements on buses and the radio yielded less-than-anticipated responses at significant cost, in terms of staff time and dollars expended. It is quite plausible that people attending large community events feel bombarded with messages and information and, therefore, disregard the new information presented. The situation may be the same on public buses and radio stations that are flooded with advertisements leading to minimal audience impact [52]. However, these venues provided a means for the MAALES Project to develop a community presence and new partnerships. Therefore, if it is possible to engage in selective community events at a relatively minimal additional cost, it could be worthwhile in terms of networking opportunities and increased study visibility. In contrast, our data argue against the utility of outreach and recruitment in bars and clubs for longitudinal studies like this that require later participation at another location. Contrary to event-based recruitment or public advertising, these settings do not provide many opportunities for networking or for repeated visibility by large numbers of people. The dark, loud, and crowded setting of many bars/clubs, coupled with the late nights and frequent intoxication of patrons may explain the poor response to outreach efforts in these settings.

Our data also show that a small but significant proportion of HIV-negative and younger men heard of the study via the Internet. These individuals could have been looking specifically to participate in research, those who saw promotional materials in the community and then went online for more information, or those who “stumbled upon” study materials while “surfing” the Internet [53]. The Internet may be an important tool for enrolling HIV-negative and younger Black MSMW into preventive studies that require in-person participation, but likely an insufficient one. For example, we acknowledge that participation and retention of higher SES individuals and younger African American MSMW in broad Internet-based studies has not been especially successful [53,54]. With the increased availability of web-based and mobile applications that makes it easier to engage in targeted recruitment efforts, the Internet may hold greater potential now for enhancing enrollment and retention efforts than it did during our outreach. These applications also have the potential to reduce the cost of recruitment efforts, as well as make it easier to remain in contact with participants, at least with those who are financially stable enough to maintain a mobile phone account and/or Internet access over time.

Much more research is needed to understand what strategies and motivations work best for encouraging participation by higher SES and younger Black MSMW who are underrepresented in this and other non-Internet based studies [34,55,56]. There are several reasons why recruitment may be challenging in these MSMW compared to lower SES and older Black MSMW. These include: a) lack of representation on the study teams, which tended to be older in our case, b) reduced concern about HIV, c) greater tradeoffs between study-associated benefits and study-related time commitments and perceived risks, d) developmental differences, many young adults may not be at a life stage that encourages the type of self-reflection and behavior change promoted in the MAALES intervention, e) study setting, the main study site was an urban university in a low-income neighborhood and two of the three CBOs were situated in areas that were also low-income, f) community partnership with CBOs that helped shape our outreach strategies and were an outreach focus, and g) formative research findings that were largely shaped by service agency consumers and service providers who worked with low-income men. Carefully designed, qualitative and rapid assessment approaches could help elucidate the relative importance of these and other potential influences on participation.

Our analyses focused on the source from which participants and others screened learned about the MAALES Project. We note, however, the types of outreach material available from these sources also varied. For example, advertisements on buses, bus benches and in newspapers were short, relative to those on condom packets, flyers, or palm cards. The lengthiest messages were in the tri-fold brochures and radio advertisements. The hand-distributed materials also tended to have images; whereas, the newspaper and Craigslist advertisements did not. The cost and format of each medium dictated what could and could not be included. It is possible that some of the differences we observed across recruitment sources were influenced by the study information content and formats associated with those sources. We also note that our investigation could have been enhanced by assessing all respondents for repeated exposure to our different outreach efforts. This may have allowed us to assess the role of multiple exposures and whether those exposed to outreach materials in multiple venues were most influenced by a particular exposure.

The underrepresentation of higher SES and younger African American MSMW reduced statistical power for assessing the relative benefits of various recruitment approaches. Middle and higher SES Black MSMW are also at elevated HIV risk and their HIV prevention needs warrant attention with greater representation. Fortunately, the data do point to some venues that may be more successful than others for reaching these subgroups. Additionally, although all recruitment efforts were carried out in Los Angeles, and mostly in south and central Los Angeles communities, many of the dynamics that drive research participation may be consistent among Black MSMW and other sexual minorities in urban U.S. settings.

## Conclusion

Our findings reiterate the crucial role that agencies play in engaging racial/ethnic minorities in research. They shed light on the importance of community engagement from start to finish and the need for creative and targeted strategies to attract diverse subpopulations of targeted groups. Despite advances in communication technology, face-to-face communication continues to play a significant role in recruiting research participants. However, current and new marketing technologies and strategies must be continually evaluated in order to assess which are best for recruiting and retaining high-risk sexual minorities.

## Figures and Tables

**Table 1:** Distribution of Field Staff Outreach and Recruitment Efforts, MAALES Project, Los Angeles, 2007–2011

Venue Type	% of Activities
**Private Businesses** *Gyms, restauran, coffee shops, bookstores, malls, Laundromats, beauty, fashion, and film schools, hair and nail salons,* *barbershops*	102 (18%)
**Agencies (Non-Governmental)** *Religious organizations, homeless shelters, homeless service centers, single resident occupancy hotels, community centers, drug treatment programs, youth centers, non-profit agencies, clinics, HIV service providers*	157 (27%)
**Agencies (Governmental)** *Government low-income housing, employment agencies, Work Source Centers, Social Security Offices, Department of Motor Vehicles, Department of Public Social Services,*	53 (9%)
**Bus/Train** *Public buses, trains (generally at stops and stations)*	44 (7%)
**Clubs and Bars** *Clubs and bars targeting MSM*	30 (5%)
**Events** *Health fairs, street festivals, free concerts, etc.*	56 (9%)
**Sex Venues** *Adult book stores, spas, cruising spots, bathhouses*	26 (4%)
**Street Outreach** *Sidewalks, street corners, college campuses, the beach, parks*	122 (21%)

**Table 2:** Source of information about study among individuals screened for enrollment, found eligible and enrolled, MAALES Project, Los Angeles, 2007–2011.

Source of Information about MAALES	Screened n=862 (% of total)	Eligible n= 491 (% of screened)	Enrolled n=437 (% of eligible)	Retained n= 291 (% of enrolled)
Agency	173 (20.1)	139 (80.3)	130 (93.5)	95 (73.1)
Bars and Clubs	11 (1.3)	4 (36.4)	4 (100.0)	4 (100)
Bus Advertisements	105 (12.2)	52 (49.5)	39 (75.0)	26 (66.7)
Event	5 (0.6)	0 (0.0)	0 (0)	0 (0.0)
Flyer (location type not specified)	135 (15.7)	48 (35.6)	41 (85.4)	24 (58.5)
Friend/Participant	171 (19.8)	101 (59.1)	92 (91.1)	60 (65.2)
Internet	56 (6.5)	31 (55.4)	21 (67.7)	14 (66.7)
Newspaper	61 (7.1)	22 (36.1)	21 (95.5)	11 (52.4)
None mentioned	36 (4.2)	17 (47.2)	16 (94.1)	13 (81.3)
Other	57 (6.6)	31 (54.4)	27 (87.1)	17 (63.0)
Radio Advertisements	6 (0.7)	3 (50.0)	3 (100.0)	2 (66.7)
Recruiter (location type not specified)	46 (5.3)	43 (93.5)	38 (88.4)	25 (65.8)
Overall %	100	57.0	89.0	66.6

5 were found ineligible following enrollment and were not followed. % Retained = Retention at 3 or 6 month follow-up, whichever was the latter.

**Table 3:** Differences in information source about the study by participant’s self-reported HIV status, MAALES Project, Los Angeles, 2007–2011.

Source of Information about MAALES	HIV-Positive n=204 (%)	HIV-Negative n=180 (%)	*p* value for difference	HIV-Positive n=204 (%)	Indet./Incon./Not Tested n=41 (%)	*p* value for difference
Agency	42.1	19.4	<.0001	42.2	19.5	.006
Bars and Clubs	0.5	1.7	0.344	0.5	0.0	1.00
Bus Ads	3.9	16.1	<.0001	3.9	4.9	0.77
Flyer (location not specified)	8.3	10.0	0.571	8.3	9.8	.761
Friend/ Participant	22.1	18.9	0.443	22.1	26.8	.506
Internet	1.5	9.4	<0.0001	1.5	2.4	.521
Newspaper	0.9	8.9	<0.0001	0.9	7.3	.034
None mentioned	4.4	1.7	0.149	4.4	9.8	.240
Other	7.4	5.6	0.476	7.4	4.9	.569
Radio Ads	0.0	1.7	0.102	0.0	0.0	--
Recruiter (location not specified)	8.8	6.7	0.431	8.8	14.6	.253

* NOTE: Chi-square tests conducted using participants with Positive status as referent group. Fisher’s Exact Test of significance used when cells contained counts less than 5.

**Table 4:** Differences in information source about the study by participants’ socioeconomic status (SES)*, MAALES Project, Los Angeles, 2007–2011.

Source of Information about MAALES	Strata Low n = 186 (%)	Strata Med n = 159 (%)	*p* value for difference	Strata Low n = 186 (%)	Strata High n = 81 (%)	*p* value for difference
Agency	34.4	27.0	0.140	34.4	24.7	0.116
Bars and Clubs	1.1	1.3	0.875	0.0	1.1	1.000
Bus Ads	5.4	10.7	0.067	5.4	13.6	0.022
Flyer (location not specified)	8.6	10.1	0.641	8.6	11.1	0.518
Friend/Participant	23.1	21.4	0.698	23.1	16.1	0.192
Internet	3.8	5.7	0.403	3.8	6.2	0.521
Newspaper	5.4	4.4	0.677	5.4	4.9	1.000
None mentioned	3.8	3.1	0.754	3.8	4.9	0.739
Other	8.8	4.8	0.141	4.8	4.9	1.000
Radio Ads	50.0	50.0	0.911	1.2	0.5	0.543
Recruiter (location not specified)	9.1	6.9	0.451	9.1	12.4	0.424

* NOTE: Strata refer to low (1), middle (2), and higher middle (3) SES based on a combination of income, education and employment status indicators. Fisher’s Exact Test of significance used when cells contained counts less than 5.

**Table 5:** Differences in information source about the study by participant age group*, MAALES Project, Los Angeles, 2007–2011.

Source of Information about MAALES	30–49 n = 264 (%)	< 30 n = 64 (%)	*p* value for difference	30–49 n = 264 (%)	50 plus n = 104 (%)	*p* value for difference
Agency	34.9	15.6	0.002	34.9	26.9	.144
Bars and Clubs	0.76	3.13	0.121	0.76	0.0	1.00
Bus Ads	8.7	7.8	0.817	8.7	10.6	.578
Flyer (location not specified)	9.9	9.4	0.908	9.9	8.7	.725
Friend/Participant	20.1	23.4	0.551	20.1	23.1	.523
Internet	2.7	14.1	0.0001	2.6	4.8	.331
Newspaper	5.7	3.1	0.401	5.7	3.9	.605
None mentioned	3.4	3.1	0.909	3.4	4.8	.550
Other	5.3	7.8	0.440	5.3	7.7	.384
Radio Ads	0.4	3.1	0.038	0.4	0.0	1.00
Recruiter (location not specified)	8.3	9.4	0.789	8.3	9.6	.694

* NOTE: Chi-square tests conducted using 30–49 age category as referent group. Fisher’s Exact Test of significance used when cell contained counts less than 5.
